# Teaching Hallucination Literacy in Medical Education: A Claim-Level Verification Framework and Toolkit

**DOI:** 10.1007/s40670-026-02907-0

**Published:** 2026-09-29

**Authors:** Hunter Colson, Hubert O. Ballard

**Affiliations:** 1 University of Kentucky College of Medicine, Lexington, KY USA; 2 Golisano Children’s at UK, Department of Pediatrics, Division of Neonatology, University of Kentucky College of Medicine and UK HealthCare, 138 Leader Avenue, Room 005, Lexington, KY 40536 USA

**Keywords:** Artificial intelligence, Large language models, Medical education, AI literacy, Critical appraisal, Patient safety

## Abstract

Generative artificial intelligence can produce fluent, persuasive responses that intermix accurate, unsupported, outdated, and fabricated information. Therefore, recommendations to simply “verify the output” are insufficient as a teachable competency. We operationalize hallucination literacy as five observable competencies: deconstructing AI responses into discrete claims, identifying required evidence, calibrating verification burden to the clinical context and consequences of error, evaluating evidence or obtaining accountable expert review, and documenting a final verification decision. The framework includes a model-agnostic verification workflow, claim verification matrix, worked example, instructional sequence, and formative assessment rubric for journal clubs, evidence-based medicine curricula, scholarly writing, and supervised clinical reasoning.

**Supplementary Information:**

The online version contains supplementary material available at https://doi.org/10.1007/s40670-026-02907-0.

## Introduction

Generative artificial intelligence (AI) has rapidly become an everyday cognitive tool for medical learners. In recent surveys, most health professions students reported using generative AI independently for learning, despite limited formal instruction on appropriate use [1, 2]. Learners increasingly use large language models (LLMs) to explain concepts, summarize literature, generate differential diagnoses, draft written work, and answer clinical questions. The educational challenge is therefore no longer whether students will encounter AI-generated information, but whether they can critically evaluate what should be trusted, verified, revised, or rejected before it influences learning or clinical reasoning.

Medical education has appropriately responded by emphasizing AI literacy, responsible use, curricular integration, faculty development, and human oversight [3–5]. These efforts provide an essential foundation for understanding how generative AI functions and how it should be incorporated into educational and clinical environments. However, they do not fully address the immediate cognitive task learners face each time they consult an AI system: determining whether an individual AI-generated claim is sufficiently supported to be accepted, cited, or acted upon. Recommendations to “double-check” or “verify” AI output acknowledge this need but rarely describe a teachable, observable, or assessable verification process. They often leave unspecified the unit of analysis, the evidence required, how verification burden should vary by context or consequence, and when independent verification should give way to accountable human supervision.

This gap has practical consequences. Fluency alone does not enable learners to recognize incorrect AI-generated information reliably. Final-year medical students correctly evaluated only a median of 56% of LLM-generated responses when accurate justification was required [6]. General practice trainees similarly identified deliberately constructed AI hallucinations with a mean accuracy of only 55%, with particularly poor performance on application- and evaluation-level clinical scenarios [7]. Citation practices add another challenge. AI systems may fabricate references, inaccurately describe legitimate sources, or cite real publications that do not support the accompanying claim [8, 9]. Fluency, professional style, citations, and hyperlinks may increase confidence, but none constitutes evidence that a claim is valid.

Rather than treating verification as a vague recommendation, we argue that it should be taught as a distinct educational competency. We operationalize *hallucination literacy* as five observable, assessable competencies: deconstructing AI-generated responses into discrete claims, identifying the type of evidence each claim requires, calibrating the burden of verification to the clinical context and potential consequences, evaluating supporting evidence or obtaining accountable expert review, and documenting a defensible verification decision.

This contribution is intentionally narrower than a comprehensive AI curriculum or clinician competency framework [4, 5, 10, 11]. It provides a practical framework that educators can teach, observe, and assess whenever learners engage with generative AI. To support implementation, Online Resource 1 includes a facilitator guide, reusable worksheet, synthetic cases with answer keys, and a formative analytic rubric.

## Why the Claim Is the Unit of Verification

For educational purposes, we use *hallucination* as an umbrella term for AI-generated content that is fabricated, unsupported, contradicted by available evidence, outdated, or inaccurately attributed. The term describes a property of the generated output rather than the mechanism that produced it. Consistent with the technical literature, hallucinations are better understood as a family of factual reliability failures than as a single phenomenon [12].

Effective verification begins with the recognition that an entire AI response is rarely the appropriate unit of evaluation. A single paragraph may simultaneously contain an accurate definition, an unsupported causal inference, an outdated clinical recommendation, and a fabricated or misrepresented citation. Labeling the response as simply “correct” or “incorrect” obscures which statements are supported, which remain uncertain, and which require correction. Verification should therefore occur at the level of the individual claim. Fine-grained factuality research has reached a similar conclusion by evaluating long-form AI outputs as collections of discrete, independently judgeable claims rather than as monolithic responses [13]. The same principle provides a natural foundation for instruction: the individual claim—not the overall response—is the appropriate unit of evidence matching, feedback, and assessment.

Claim-level verification also limits inappropriate transfer of credibility. Once learners confirm one statement in a response, they may inadvertently extend that confidence to neighboring statements that have not been independently evaluated. Deconstructing a response into discrete claims interrupts this halo effect by requiring each proposition to earn its own evidentiary support. This distinction is particularly important for recommendations, which often combine multiple independently verifiable claims: the intervention, target population, relevant clinical circumstances, dosing or safety considerations, and applicability to the patient or setting under consideration.

Viewing verification through the lens of individual claims transforms hallucination literacy from a general attitude of skepticism into observable, assessable competencies. Learners demonstrate these competencies by deconstructing AI-generated responses into discrete claims, identifying the appropriate verification target for each claim, calibrating verification burden to the relationship between the claim, available evidence, and potential consequences of error, evaluating supporting evidence or obtaining accountable expert review when appropriate, and documenting a defensible verification decision with any remaining uncertainty. These behaviors provide concrete targets for instruction, feedback, coaching, and assessment.

## Verification Targets

Not every AI-generated claim requires the same type of verification. Before learners can determine whether a claim is trustworthy, they must identify *what kind of claim it is*, because the appropriate evidence depends on the nature of the question being asked. Classifying the verification target bridges claim identification and evidence evaluation. Rather than encouraging indiscriminate fact-checking, it directs learners toward the evidence most capable of resolving uncertainty. We group verification targets into four educationally useful families. The first is **factuality**, which asks whether a claim is true within the relevant scientific or clinical context. Factuality checks may involve mechanisms of disease, diagnostic criteria, numerical results, medication doses, adverse effects, or treatment recommendations.

The second family is source support, which combines the related concepts of faithfulness and attribution. Both require learners to compare a claim directly against the source that the AI system was instructed to summarize, cite, or reference. A statement may be factually correct yet unfaithful if the cited article did not make that claim. Likewise, a genuine citation may be irrelevant, insufficient, or overstated. Research on abstractive summarization and attribution has similarly emphasized that the presence of a citation is not itself evidence of support; the cited source must substantiate the specific claim being made [14, 15]. For instructional purposes, faithfulness and attribution can be taught within a single source-support category while preserving the distinction between unsupported summaries and unsupported citations.

The third family addresses recency and locality. Some claims depend not only on factual accuracy but also on whether the information reflects current clinical guidance and applies within the learner’s jurisdiction or institution. A recommendation may accurately reflect a previous guideline yet have been superseded, or it may be evidence-based but inconsistent with local formularies, institutional policies, or regional regulations. The fourth family is patient-specific applicability, which asks whether a generally accurate statement is appropriate for an individual patient. AI-generated outputs frequently shift from general education to patient-specific recommendations involving diagnosis, treatment, dosing, triage, documentation, or other consequential decisions. Determining whether such recommendations are appropriate often requires integrating multiple verification targets, including factual accuracy, current evidence, contraindications, patient preferences, local policies, and appropriate clinical supervision.

Although these target families often overlap, they are not interchangeable. Consider the statement: *“The uploaded trial demonstrated a mortality benefit*,* so Drug X should be first-line therapy for this patient.”* Evaluating this recommendation requires multiple forms of verification. Learners must determine whether the cited trial supports the stated finding (source support), whether the claim remains consistent with current evidence and clinical guidelines (factuality and recency), and whether the recommendation applies to the patient’s clinical circumstances (patient-specific applicability). Classification is therefore not merely labeling; it determines which evidence is needed to evaluate a claim appropriately.

This distinction remains important even when AI systems provide citations or use retrieval-augmented generation. Retrieval may improve access to external information and increase transparency regarding source provenance [16], but it cannot ensure that the retrieved evidence is relevant, current, correctly interpreted, or sufficient to support the generated claim [17, 18]. Consequently, learners should not ask only, *“Did the model provide a source?”* They should ask, *“Is this the appropriate source*,* is it current and authoritative*,* and does the inspected evidence support this specific claim?”*

## The Claim-Level Verification Workflow

**Fig. 1:**
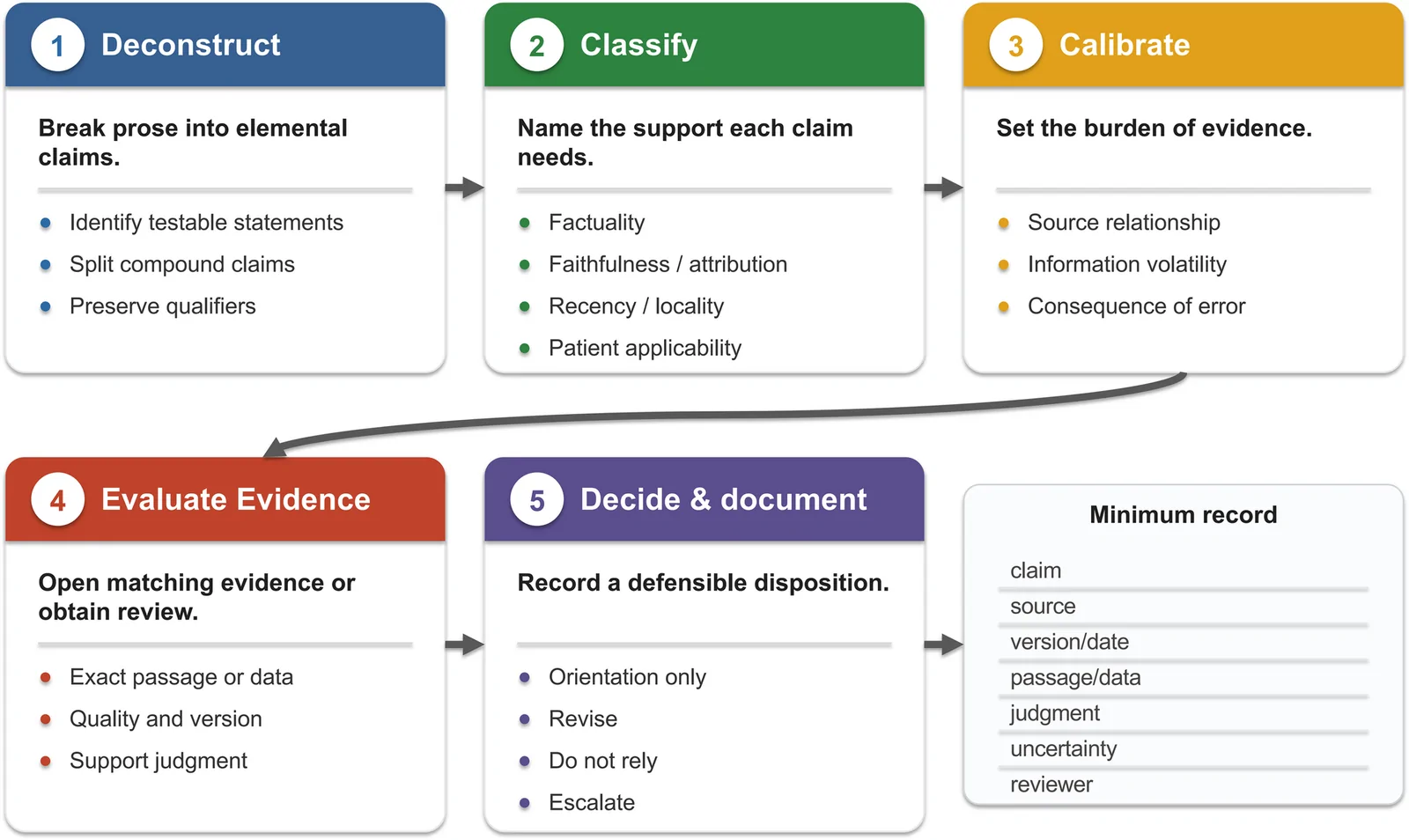
Claim-level verification workflow for generative AI output. The learner deconstructs a response into elemental claims; classifies factuality, faithfulness/attribution, recency/locality, and patient-specific applicability targets; calibrates the burden of evidence according to source relationship, information volatility, and consequence; inspects matching evidence or obtains accountable review; and records a disposition and verification record. Orientation only does not authorize transfer into patient care, graded assessment, formal documentation, or scholarly publication without independent verification

The claim-level verification workflow turns verification from an intuitive judgment into an observable, reproducible process that can be taught, coached, and assessed. Instead of asking learners simply to “check the answer,” it defines five sequential competencies: deconstructing AI-generated responses into discrete claims, identifying the appropriate verification target, calibrating verification burden, evaluating supporting evidence, and documenting a defensible decision (Fig. 1).

Although presented sequentially, the workflow is iterative; learners may revisit earlier stages as claims evolve or uncertainty persists. Because the framework evaluates human verification behavior rather than model performance, it applies across a wide range of AI systems, including chatbots, retrieval-augmented tools, electronic health record integrations, and emerging agentic interfaces.

### Step 1: Deconstruct

Verification begins by deconstructing an AI-generated response into discrete, independently verifiable claims. Learners should identify each consequential proposition that could reasonably be judged true or false, supported or unsupported, current or outdated, or applicable or inapplicable. Compound statements should be separated whenever different clauses require different evidence. Qualifiers—including patient population, intervention, comparator, timing, dose, and degree of certainty—should remain attached to the claim because they frequently determine the verification target and evidence required. The goal is not to fragment every sentence mechanically, but to ensure that every meaningful claim can be evaluated on its own merits.

One useful heuristic is to ask whether one portion of a sentence could be supported while another could be unsupported. If so, the sentence contains more than one claim and should be separated before verification proceeds.

### Step 2: Classify

Once claims have been identified, learners classify one or more verification targets for each claim. This step determines the evidence needed before verification begins. Rather than encouraging indiscriminate fact-checking, classification directs learners toward the most appropriate evidence and makes subsequent source selection transparent and explainable. As described above, verification targets may include factuality, source support, recency and locality, patient-specific applicability, or a combination of these domains.

### Step 3: Calibrate

Not every claim requires the same level of evidence before it can reasonably be relied upon. We refer to this evidentiary threshold as the **verification burden**: the minimum level of supporting evidence and accountable review required before a learner relies on an AI-generated claim.

**Fig. 2:**
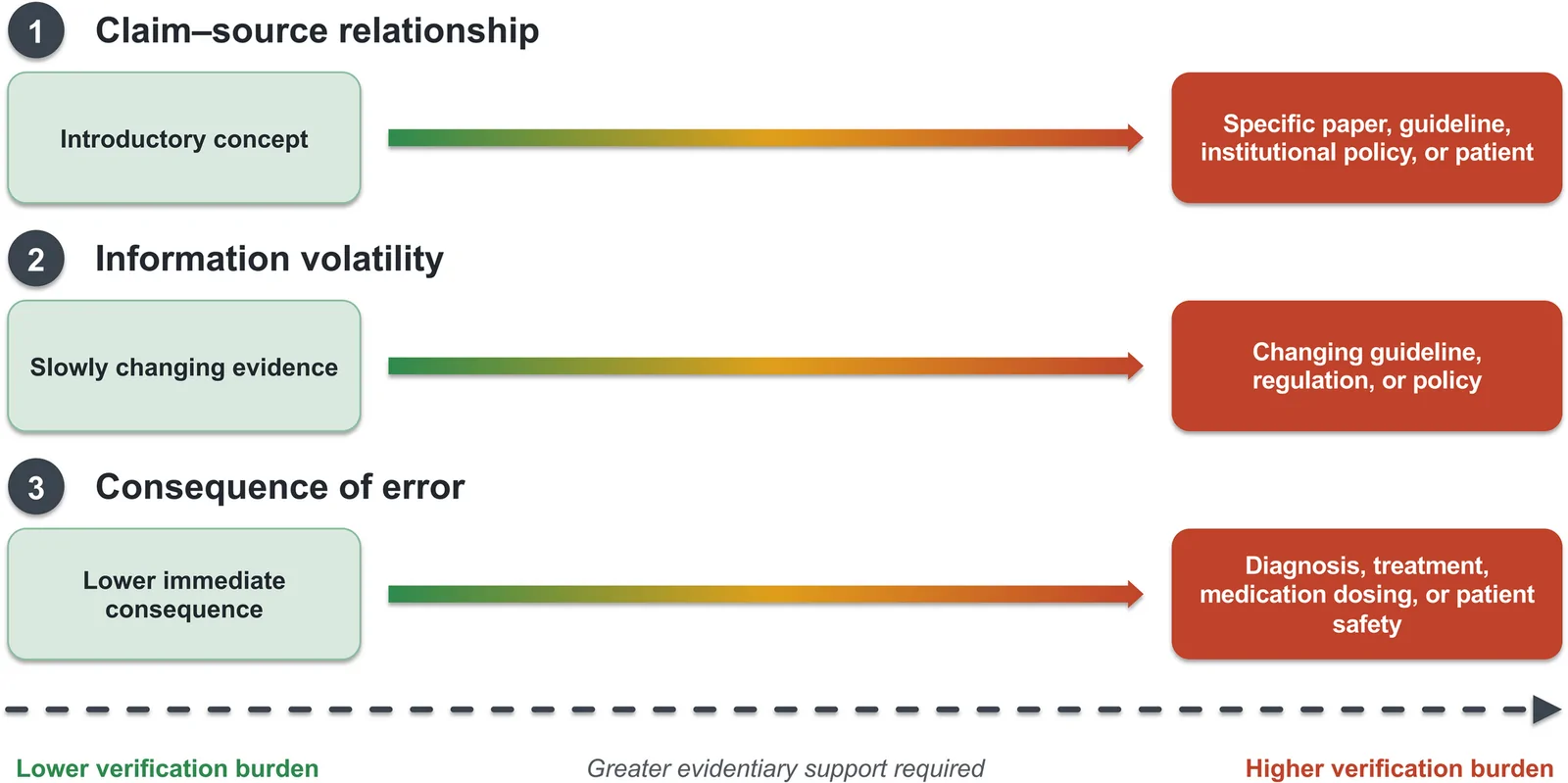
Verification-burden spectrum across three dimensions. Verification burden increases from introductory concepts to source- or patient-specific assertions, from slowly changing evidence to changing guidance, and from lower immediate consequences to decisions affecting patient safety. Learner expertise and potential consequences for later learning also inform the burden of evidence and review required before use

Verification burden increases along three dimensions (Fig. 2). The first is the claim’s relationship to its source: an introductory explanation may require less extensive checking than a claim about a specific study or patient. Familiarity alone does not establish support, and novice learners may need faculty guidance to identify what requires checking. The second is information volatility, from slowly changing evidence to frequently revised guidance. The third is the consequence of error. Educational explanations may have fewer immediate clinical consequences, yet misconceptions can affect later reasoning. Verification burden should therefore reflect both the intended use and the potential harm of carrying an error forward.

Verification burden concerns the evidence required before reliance, not the probability of a model error. Low-burden claims may support orientation after confirmation with an appropriate educational source. Moderate-burden claims require direct inspection of supporting evidence. High-burden claims require appropriate authoritative evidence, explicit acknowledgment of uncertainty, and accountable review by a supervisor or domain expert.

### Step 4: Evaluate Evidence

Evidence evaluation requires matching sources to the claim and appraising their quality. For introductory concepts, use current course materials, textbooks, or reviews with identifiable authorship and supporting references. Evaluate the evidence’s methods, assumptions, limitations, and representation of relevant populations. Agreement between sources may reflect shared evidence or biases rather than independent corroboration. Guideline recommendations require the issuing organization’s current publication; source-specific summaries require the original methods, results, tables, limitations, and supplementary materials. Medication recommendations require drug references and patient-specific review, while institutional workflows require current local policies. For source-linked AI output, inspect the original passage rather than the model’s paraphrase. When evidence is incomplete or conflicting, document the uncertainty and seek review appropriate to the claim.

The outcome of evidence evaluation is a support judgment: directly supported, supported with qualification, extrapolatory, contradicted, not addressed, or source unavailable. These judgments describe support within the inspected evidence and its limitations. Agreement with a source does not by itself establish factual accuracy. A claim not addressed by a source remains unsupported by it, even if plausible, and inability to retrieve a source does not establish fabrication.

Table 1 outlines the minimum supporting evidence expected for common educational uses of generative AI and the escalation triggers that warrant accountable human review. These recommendations are intended to guide educational practice rather than replace clinical judgment.

**Table 1:** Verification burden and minimum supporting evidence by generative AI use case

Use case	Primary target	Minimum supporting evidence	Escalation trigger (when to seek accountable review)
Introductory concept or explanation	Factuality	Check consequential details against a current course source, textbook, or review with identifiable evidence; use for orientation only.	Unfamiliar, contested, long-tail [19], examination-critical, or practice-critical content.
Source-specific summary	Faithfulness	Compare each claim with the source methods, results, tables, limitations, and supplements; mark model-added inferences.	Causal, clinical, or practice implication not stated in the source; ambiguous or incomplete passage.
Citation or provenance request	Attribution	Confirm source existence, metadata, correction or retraction status, and passage-level support for the exact claim.	Missing, inaccessible, corrected, retracted, irrelevant, or nonsupporting source.
Current guideline or local policy	Factuality; recency/locality	Open the issuing society, regulator, public-health agency, or institution; confirm population, version, and date.	Conflicting or superseded guidance; local implementation or policy dependence.
Medication or dosing question	Factuality; patient applicability	Use an authoritative drug source [20]; verify indication, dose, organ function, interactions, contraindications, pregnancy, and age.	Any high-risk patient factor, interaction, contraindication, or uncertain dose.
Patient-specific reasoning	Factuality; applicability; consequence	Use output only to organize the question; independently verify actionable claims under clinician supervision and discuss goals, preferences, and practical constraints with the patient.	Any diagnosis, treatment, triage, dosing, documentation, or other safety-critical decision.
Source-linked or retrieval-augmented output	Faithfulness; attribution	Inspect the original passage and source quality, date, and scope; check for unsupported extrapolation.	Irrelevant, outdated, weak, contradictory, or overextended retrieved evidence.

Note. Verification burden is the evidentiary burden before reliance: the minimum supporting evidence and accountable review required before a learner may use a claim. Orientation only never authorizes patient care, graded assessment, formal documentation, or scholarly publication without independent verification

### Step 5: Decide and Document

The final step converts evidence evaluation into an explicit verification decision. Each claim receives one of four dispositions: Orientation only, Revise, Do not rely, or Escalate. *Orientation only* indicates that the claim may support low-stakes learning but should not be transferred into patient care, graded assessment, formal documentation, or scholarly publication without independent verification. *Revise* indicates that the claim can be corrected, narrowed, qualified, or appropriately reattributed to align with the supporting evidence. *Do not rely* indicates that available evidence is insufficient, contradictory, inaccessible, or otherwise inadequate for the intended use. *Escalate* indicates that independent verification has reached its appropriate limit and that accountable review by a supervisor, pharmacist, librarian, specialist, or institutional policy owner is required before further reliance.

Verification concludes with documentation sufficient for another individual to reproduce the reasoning process. At a minimum, learners should record the claim evaluated, the supporting source or authority, the relevant version or publication date when applicable, the specific evidence inspected, the support judgment, remaining uncertainty, and the final disposition. Documentation is more than record keeping; it is the mechanism by which verification becomes observable. Recording these elements transforms a private cognitive process into an inspectable educational performance that can be discussed, reproduced, coached, and assessed. This emphasis on transparent decision making aligns with broader principles of trustworthy AI, accountable human oversight, and calibrated reliance rather than unquestioning acceptance or blanket skepticism [21–23].

### Worked Example

Consider the synthetic statement: “The 2026 guideline recommends Drug X as first-line therapy for all adults with Condition Q. Drug X is safe in renal impairment and requires no dose adjustment, so it should be started today for this patient.” Drug X and Condition Q are fictional; the example is not clinical guidance.

Rather than a single assertion, the statement contains multiple independent claims that require separate verification. These include the existence of a current guideline, the recommendation of Drug X as first-line therapy, the applicability of that recommendation to all adults with Condition Q, the absence of renal dosing restrictions or safety concerns, and the recommendation to initiate treatment for an individual patient. As shown in Table 2, each claim maps to a different verification target and may require different evidence sources and verification actions.

This example shows why the instruction to “check the answer” is insufficient. A guideline may identify a recommended therapy and population while referring readers elsewhere for dosing. A drug monograph may address renal dosing but cannot establish whether treatment fits a patient’s clinical circumstances, goals, preferences, or practical constraints. Those questions require discussion with the patient and clinical judgment. Verification must therefore occur at the level of individual claims. Even when every component fact is supported, the final recommendation requires consideration of whether the patient wants the treatment and can undertake it.

**Table 2:** Claim-level decomposition of a synthetic medication recommendation

Elemental claim	Verification target(s)	Evidence action	Disposition
A 2026 guideline recommends Drug X.	Factuality; recency	Verify the issuing body, version, date, and exact recommendation.	Revise or do not rely if the claim is unsupported or superseded.
Drug X is first-line therapy for all adults with Condition Q.	Faithfulness; factuality; population	Inspect recommendation wording, eligibility, exclusions, and strength.	Narrow to the supported population or do not rely.
Drug X is safe in renal impairment.	Factuality	Use an authoritative drug source; distinguish general use from dose-specific safety.	Revise or do not rely on an overbroad statement.
No dose adjustment is needed at this renal function.	Factuality; patient applicability; consequence	Verify the exact renal threshold, indication, dose, interactions, and patient factors.	Escalate; do not rely on generated output alone.
Drug X should be started today for this patient.	Patient applicability; locality; consequence	Review clinical factors, medications, local policy, and the patient’s goals and preferences with the supervisor and patient.	Escalate before any patient-specific action.

Note. Drug X, Condition Q, and all dates and thresholds are fictional; the example is educational and is not clinical guidance

### Teaching Hallucination Literacy

The claim-level verification workflow is designed for integration into existing medical education activities rather than delivery as a stand-alone AI curriculum. Because the framework emphasizes observable verification behaviors rather than technical knowledge of AI systems, it can be incorporated wherever learners already evaluate evidence, interpret literature, or make clinical decisions.

In journal clubs, learners can compare AI-generated summaries with the original article’s methods, results, tables, and limitations. During evidence-based medicine instruction, they can identify individual claims and determine the evidence required to support each recommendation. In scholarly writing, learners can verify AI-suggested references for existence, bibliographic accuracy, and claim-specific support while maintaining human responsibility for the final manuscript [24]. Clinical reasoning conferences provide opportunities to identify which claims require guidelines, drug references, institutional policies, accountable supervision, or a decision not to rely on the AI-generated output.

Instruction should progress from structured to increasingly authentic verification tasks. Early learners benefit from curated source sets that allow them to practice claim deconstruction, identify verification targets, and distinguish among factuality, source support, recency, and patient-specific applicability without the additional challenge of locating evidence. As learners gain experience, they can independently identify authoritative sources, evaluate source quality, check whether the evidence remains up to date, reconcile conflicting recommendations, and explain remaining uncertainty.

Advanced learners, including residents and practicing clinicians, can apply the framework to complex cases involving medications, institutional policies, and patient-specific decisions that require explicit escalation and accountable human review. This progression reflects the educational goal of gradually transferring responsibility while maintaining appropriate supervision. Consistent with prior work in medical education, these activities embed AI critique within existing content instruction rather than treating it as a separate technical skill [25].

The quality of instruction depends heavily on case design. Effective cases should intentionally combine multiple verification challenges rather than present obviously correct or incorrect answers. A single case might include supported factual claims, unsupported inferences, attribution errors, outdated guidance, locality-dependent recommendations, and at least one patient-specific claim requiring escalation. Cases generated in real time should not be used without prior faculty review of both the AI-generated response and the supporting evidence. Likewise, protected health information should never be entered into unapproved AI systems, and synthetic educational cases should remain clearly distinguished from clinical guidance.

The framework is intended to support active discussion rather than passive fact-checking. A typical 60-minute session may include a brief orientation, individual claim deconstruction, evidence evaluation, small-group comparison of verification decisions, and a facilitated debrief focused on the evidence that changed each learner’s judgment. Longer sessions can introduce conflicting authoritative sources, superseded guidelines, or plausible but unsupported inferences to increase complexity and encourage discussion of uncertainty and escalation decisions.

To facilitate implementation, Online Resource 1 includes a ready-to-use facilitator guide, session plans, quick-reference materials, a learner claim ledger, synthetic cases with answer keys, and a formative analytic rubric that can be incorporated directly into existing educational activities.

### Formative Assessment

A central contribution of the claim-level verification framework is that it makes AI verification observable. Rather than treating safe AI use as an internal judgment or as an outcome inferred only from a correct answer, the framework defines a sequence of verification behaviors that educators can directly observe, discuss, and assess. Assessment therefore shifts from asking whether a learner arrived at the correct conclusion to evaluating whether the learner followed a transparent and reproducible verification process.

The accompanying analytic rubric operationalizes this process as five observable competencies: deconstructing AI-generated responses into discrete claims, identifying the appropriate verification target for each claim, calibrating verification burden, evaluating supporting evidence while acknowledging remaining uncertainty, and selecting an appropriate verification disposition with documentation and escalation when necessary.

These competencies reflect the reasoning process underlying safe AI use rather than the correctness of any single response. Consequently, a learner who reaches the correct conclusion without identifying the relevant claims, selecting appropriate evidence, or documenting a defensible verification pathway has demonstrated a different level of performance than a learner whose reasoning can be independently inspected and reproduced. The rubric is intended to support formative assessment, coaching, and feedback rather than high-stakes evaluation.

Because each stage of the verification workflow is directly observable, educators can identify specific patterns of learner performance that would otherwise remain hidden. For example, learners may consistently overlook consequential claims during deconstruction, confuse the existence of a citation with evidence that supports the associated claim, rely on outdated or nonauthoritative sources, extend conclusions beyond the available evidence, or fail to escalate uncertainty appropriately. These behaviors provide concrete targets for feedback while allowing programs to monitor the development of verification competencies over time.

Although the framework is designed for formative use, it also provides a foundation for future assessment research. Programs wishing to adopt the rubric for high-stakes decisions would require additional evidence supporting content validity, response processes, internal structure, relationships to other variables, consequences of testing, rater training, interrater reliability, and standard setting. Establishing such evidence is beyond the scope of this work but represents an important direction for future scholarship.

Documentation is the final observable product of the verification process. A concise verification statement might read: *“I used the AI system to organize the question. Claims 1 and 2 were verified using [source*,* version*,* and relevant passage]. Claim 3 was not supported by the available evidence. Claim 4 was escalated to [reviewer] because it could influence patient care.”* The purpose of this documentation is not administrative record keeping. Rather, it makes AI reliance transparent, transforms private reasoning into an inspectable educational performance, and provides a reproducible basis for feedback, coaching, and assessment.

### Implementation and Safety Boundaries

This framework is intended to complement, rather than replace, existing institutional policies governing AI use. Successful implementation requires clear expectations regarding approved AI systems, data governance, privacy, supervision, and acceptable educational use. The framework should not be interpreted as authorization to enter protected health information into external systems or to bypass institutional requirements for documentation, assessment, or clinical decision making. When appropriate evidence cannot be accessed, the appropriate educational response is not to substitute weaker evidence, but to acknowledge the limitation and seek accountable review.

Because the approach evaluates learner behavior rather than AI system performance, implementation should remain model-agnostic. Interfaces, retrieval capabilities, citation displays, and model architectures will continue to evolve, but the educational task of matching individual claims to appropriate supporting evidence is comparatively stable. Programs should therefore define verification expectations in terms of evidence standards rather than specific AI products or features.

Faculty calibration is essential for consistent implementation. Educators should establish shared expectations for acceptable evidence in common learning activities so that comparable claims receive comparable verification standards across instructors, courses, and clinical sites. High verification burdens should be reserved for claims whose source relationship, information volatility, or potential consequences justify additional scrutiny, thereby reinforcing calibrated rather than indiscriminate verification.

Implementation can begin within existing educational activities rather than through new courses. For example, a journal club may ask learners to annotate an AI-generated summary, identify the claim most likely to alter interpretation of the study, and locate the passage that supports or refutes it. As learners gain experience, cases can progressively incorporate unsupported citations, outdated recommendations, locality-dependent guidance, patient-specific medication decisions, or retrieved passages that invite unsupported extrapolation. Across these activities, the educational objective remains consistent: learners should be able to justify why a claim was used only for orientation, revised, not relied upon, or escalated for accountable review.

Completion of the verification process should not be interpreted as proof that evidence is complete, unbiased, or sufficient to support patient care. Human judgment remains essential whenever claims involve individualized clinical decisions, safety-critical recommendations, institutional policies, or other contexts in which uncertainty carries meaningful consequences.

### Limitations and Research Agenda

This work presents an evidence-informed educational model rather than a validated assessment instrument. It does not estimate hallucination prevalence, compare AI systems, certify model reliability, or replace institutional governance. The supporting literature spans multiple model generations, prompting strategies, specialties, and educational settings, all of which will continue to evolve as generative AI technologies advance. Although *hallucination* is used throughout this paper as a practical educational umbrella, it encompasses multiple forms of factual reliability failure rather than a single technical phenomenon.

Effective verification requires domain knowledge, information retrieval skills, source access, and time. The full workflow may be impractical during urgent clinical care and must not delay necessary treatment or supervision. Claim deconstruction may increase cognitive load, particularly for novices. Verification burden categories are pragmatic rather than psychometrically derived, and institutions may adopt different escalation thresholds according to their educational goals and supervision structures. Implementation should avoid excessive checking of inconsequential claims or unnecessary distrust of AI-generated information.

These limitations define an agenda for future research. Prospective studies should evaluate whether this approach improves learners’ ability to identify unsupported claims, distinguish source existence from source support, detect consequential errors, calibrate reliance, select authoritative evidence, and make appropriate escalation decisions compared with conventional advice to simply “verify AI output.” Additional work should examine transfer across undergraduate medical education, residency, and continuing professional development; develop evidence supporting assessment validity; evaluate faculty calibration and rater agreement; and identify unintended consequences, including inequitable access to authoritative sources, increased learner workload, and potential displacement of foundational learning.

## Conclusion

Generative AI has transformed verification from an occasional scholarly task into a routine educational competency. Treating AI-generated responses as either wholly trustworthy or wholly unreliable obscures the fact that accurate, unsupported, outdated, and fabricated claims often coexist within the same fluent response. Effective verification therefore begins by shifting the unit of judgment from the overall response to the individual claim.

This paper proposes claim-level verification as a practical framework for operationalizing hallucination literacy in medical education. By deconstructing AI-generated responses into discrete claims, identifying the appropriate verification target, calibrating verification burden, evaluating supporting evidence, and documenting a defensible verification decision, learners demonstrate a sequence of observable competencies that educators can teach, coach, and assess. Rather than encouraging blanket skepticism or uncritical acceptance, the framework promotes calibrated reliance grounded in transparent evidence evaluation and accountable human oversight.

As generative AI becomes increasingly integrated into medical education and clinical practice, the ability to verify AI-generated information may become as fundamental as searching the literature or appraising evidence. This approach offers a practical way to make that competency explicit, teachable, and assessable while future research continues to refine and validate its implementation.

## Supplementary Information

Below is the link to the electronic supplementary material.

Supplementary Material 1 (DOCX 50.4 KB)

## Data Availability

No datasets were generated or analyzed. The educational materials are provided in Online Resource 1.

## Declarations

### Use of generative AI

During manuscript preparation, the authors used OpenAI ChatGPT and Codex to support literature-query development, language and organizational revision, consistency checking, and code-assisted document and figure production. Generative AI was not treated as a source of scientific authority or listed as an author. The authors independently inspected and verified all claims, references, educational materials, and artwork, and accept full responsibility for the integrity and accuracy of the work.
