# Supplementary material for: Teaching Hallucination Literacy in Medical Education: A Claim-Level Verification Framework and Toolkit
Source: Med Sci Educ. 2026 Sep 29;36(6):2747–56. doi: 10.1007/s40670-026-02907-0 (PMC13759939; doi:10.1007/s40670-026-02907-0)
Supplement: Supplementary file 1 [file 40670_2026_2907_MOESM1_ESM.docx]

**Online Resource 1**

**Claim-Level Hallucination Literacy Teaching Toolkit for Medical Educators**

**Accompanying article: Teaching Hallucination Literacy in Medical Education: A Claim-Level Verification Framework and Toolkit
Journal:** Medical Science Educator

**Authors:** Hunter Colson, BS; Hubert O. Ballard, MD

**Affiliations:** University of Kentucky College of Medicine; Golisano Children’s at UK, Department of Pediatrics, Division of Neonatology, University of Kentucky College of Medicine and UK HealthCare

**Corresponding author:** Hubert O. Ballard, MD, Hubie.Ballard@uky.edu

**Educational-use statement.** All cases, sources, organizations, drugs, policies, thresholds, and citations in this resource are synthetic unless explicitly stated otherwise. They are provided for education only and are not clinical guidance. Do not use any example in this toolkit to guide patient care, medication decisions, institutional policy, scholarly citation, or assessment without independent verification and appropriate supervision.

# 1. Overview and Scope

This resource supports implementation of the claim-level hallucination literacy framework described in the accompanying article. It is designed for use in undergraduate medical education, graduate medical education, continuing professional development, journal clubs, evidence-based medicine sessions, scholarly writing instruction, and supervised clinical reasoning activities.

The toolkit is a practical teaching resource rather than a test of AI model performance. Learners evaluate AI-generated text at the level of individual claims, match each claim to the evidence required to verify it, calibrate the burden of verification to the context and potential consequences, inspect the relevant evidence or obtain accountable review, and document a defensible disposition.

Educators may use the materials as written, adapt the synthetic cases, substitute local source sets, or ask advanced learners to locate authoritative sources independently. Before use, faculty should align the activity with local policies governing approved AI tools, data privacy, learner supervision, documentation, and clinical decision making.

# 2. Learning Objectives

After completing the activity, learners should be able to:

- Deconstruct AI-generated output into discrete, independently verifiable claims while preserving consequential qualifiers such as population, intervention, comparator, timing, dose, setting, and certainty.
- Classify the verification target for each claim as factuality, source support (faithfulness/attribution), recency/locality, patient-specific applicability, or a combination of targets.
- Calibrate verification burden using the claim-source relationship, information volatility, and consequence of error.
- Select current evidence appropriate to the claim and evaluate its provenance, methods, population, limitations, and possible biases.
- Distinguish source existence from claim-specific support and classify support as direct, qualified, extrapolatory, contradicted, not addressed, or unavailable.
- Choose an appropriate disposition for each claim: Orientation only, Revise, Do not rely, or Escalate.
- Document a reproducible verification record that includes the claim, source, version or date, inspected passage or data, support judgment, uncertainty, disposition, and reviewer when applicable.

# 3. Quick-Reference Guide

Use this section as a one-page orientation for learners before they begin the cases. The terms and disposition options mirror the accompanying manuscript.

| **Workflow step** | **Learner action** | **Prompt to ask** |
| --- | --- | --- |
| 1. Deconstruct | Break the AI-generated response into elemental claims. | Could one part of this sentence be supported while another part is unsupported? |
| 2. Classify | Identify the verification target(s) for each claim. | What kind of evidence would resolve uncertainty about this claim? |
| 3. Calibrate | Set the verification burden before relying on the claim. | How specific, changeable, or consequential is the claim if wrong? |
| 4. Evaluate evidence | Inspect matching evidence or obtain accountable review. | Does the evidence support this exact claim, and how do its quality, assumptions, and limitations affect that judgment? |
| 5. Decide and document | Record a support judgment, disposition, uncertainty, and escalation route. | What can be used, revised, rejected, or escalated, and why? |

| **Verification target** | **Question being asked** | **Appropriate evidence examples** | **Common pitfall** |
| --- | --- | --- | --- |
| Factuality | Is the claim true in the relevant scientific or clinical context? | Current course materials, textbooks, review articles, guidelines, drug references, original studies, or authoritative clinical resources. | Accepting fluent or familiar-sounding text without checking consequential details. |
| Source support (faithfulness/attribution) | Does the cited, uploaded, retrieved, or summarized source support the exact claim? | Original article methods, results, tables, figures, limitations, supplementary materials, citation metadata, correction/retraction status, and passage-level evidence. | Treating a real citation or retrieved passage as proof that the attached claim is supported. |
| Recency/locality | Is the information current and applicable to the relevant jurisdiction, institution, or course? | Issuing society, regulator, public-health agency, institutional policy, local formulary, or current course guidance, with date and version checked. | Using a superseded guideline or a nonlocal policy as if it applies universally. |
| Patient-specific applicability | Does a generally accurate statement apply to this patient or decision? | Patient-specific clinical data, contraindications, medication reconciliation, discussion of goals and preferences with the patient, practical constraints, local workflow, and supervisor or specialist review. | Moving from general education to diagnosis, treatment, dosing, triage, or documentation without accountable review. |

| **Support judgment** | **Use when** |
| --- | --- |
| Directly supported | The inspected evidence supports the exact claim, including key qualifiers. |
| Supported with qualification | The evidence supports a narrower, more conditional, or more uncertain version of the claim. |
| Extrapolatory | The claim extends beyond the evidence but is not directly contradicted by the inspected source. |
| Contradicted | The inspected evidence conflicts with the claim. |
| Not addressed | The source does not evaluate the claim, even if the claim remains plausible. |
| Source unavailable | The required source cannot be retrieved, inspected, or authenticated. |

| **Disposition** | **Meaning** | **Boundary** |
| --- | --- | --- |
| Orientation only | The claim may be used to guide low-stakes learning or organize a question after appropriate confirmation. | Does not authorize patient care, graded assessment, formal documentation, or scholarly publication without independent verification. |
| Revise | The claim can be corrected, narrowed, qualified, or reattributed so that it matches the supporting evidence. | The revised claim should be rechecked against the source before further use. |
| Do not rely | Available evidence is insufficient, contradictory, inaccessible, outdated, irrelevant, or otherwise inadequate for the intended use. | Do not use the claim as evidence, clinical guidance, or a citation. |
| Escalate | Independent verification has reached its appropriate limit and accountable review is required. | Use a supervisor, pharmacist, librarian, specialist, institutional policy owner, or other accountable reviewer as appropriate to the claim. |

# 4. Facilitator Guide

## Preparation Checklist

- Select one or more cases and decide whether learners will receive a curated source set or locate sources independently.
- Verify every planted error, unsupported inference, source mismatch, outdated recommendation, and escalation trigger before the session.
- Define approved AI tools, approved information resources, protected-health-information rules, and the local route for accountable escalation.
- Decide whether the activity will be used for formative feedback only. The rubric in this toolkit has not been validated for high-stakes decisions.
- For novice learners, provide source excerpts before asking them to perform open-ended searching; for advanced learners, progressively remove scaffolding.
- Remove or rewrite any example that could be mistaken for advice about a real patient, drug, policy, course examination, institution, or clinical workflow.
- Calibrate facilitators by reviewing the answer key together and agreeing on acceptable evidence, support judgments, dispositions, and escalation thresholds.

## Materials to Distribute

- Quick-reference guide and definitions of verification targets, support judgments, and dispositions.
- One generated-output case with its source set, or an approved list of resources if learners are expected to search independently.
- Learner claim ledger from Section 5.
- Local privacy, data-governance, and AI-use expectations, including explicit prohibition on entering protected health information into unapproved systems.
- Analytic rubric if learners will receive structured formative feedback.

## Suggested 60-Minute Session

| **Time** | **Activity** | **Facilitator focus** |
| --- | --- | --- |
| 0-10 min | Prebrief | Define claim-level verification, verification targets, verification burden, support judgments, and the four dispositions. |
| 10-20 min | Individual claim deconstruction | Learners mark independently verifiable clauses, preserve key qualifiers, and assign preliminary verification targets. |
| 20-40 min | Evidence inspection | Learners inspect supplied or approved sources, quote or paraphrase passage-level evidence, and record support judgments. |
| 40-50 min | Small-group decision | Learners compare dispositions, identify the claim most consequential if wrong, and name claims requiring escalation. |
| 50-60 min | Facilitated debrief | Faculty ask which evidence changed each judgment, highlight source-claim mismatches, and reinforce safety boundaries. |

## Suggested 90-Minute Extension

Add a second case that includes conflicting authoritative sources, a superseded version, a locality-dependent recommendation, or a plausible model-generated inference not stated in the source. Ask learners to document residual uncertainty and identify the person, service, or policy owner to whom they would escalate before using the claim outside the teaching exercise.

## Debrief Questions

- Which sentence contained more than one claim, and what changed after it was split?
- Did any real-looking source fail to support the claim attached to it?
- What supports this source's authority, which populations does its evidence represent, and what assumptions or limitations affect this claim?
- How did the verification burden change when recency, locality, or patient consequence was considered?
- Which claim could be used only for orientation, and what would make even orientation-only use inappropriate?
- Which uncertainty required accountable human review rather than additional independent searching?
- Where did domain knowledge, information-retrieval skill, source access, or tool design limit performance?

# 5. Learner Claim Ledger

**Instructions for learners:** For each consequential claim, document the verification target, burden, source and passage, support judgment, uncertainty, disposition, and escalation plan. Include source limitations or shared assumptions that affect the judgment. Evaluate each claim before judging the answer as a whole.

**Support judgments:** directly supported; supported with qualification; extrapolatory; contradicted; not addressed; source unavailable. **Dispositions:** Orientation only; Revise; Do not rely; Escalate. Support judgments describe how a claim relates to the inspected evidence and its limitations.

| **Atomic claim** | **Target(s)** | **Burden rationale** | **Source / version / passage** | **Support judgment** | **Disposition / uncertainty / escalation** |
| --- | --- | --- | --- | --- | --- |
|  |  |  |  |  |  |
|  |  |  |  |  |  |
|  |  |  |  |  |  |
|  |  |  |  |  |  |
|  |  |  |  |  |  |
|  |  |  |  |  |  |
|  |  |  |  |  |  |
|  |  |  |  |  |  |

After completing the ledger, write a concise verification statement: (1) identify the claim most consequential if wrong, (2) name the source or reviewer required before the claim could be used outside the exercise, and (3) state the final disposition.

# 6. Synthetic Practice Case 1: Source Support and Faithfulness

**Task:** Compare the generated summary with the supplied fictional article excerpt. Deconstruct the summary into claims, classify each verification target, inspect the source excerpt, and document a disposition for each claim.

## Fictional Source Excerpt

A single-center randomized pilot study enrolled 84 adults discharged after hospitalization for a fictional chronic symptom syndrome. A structured medication-counseling intervention improved the 8-week symptom score by 2.1 points compared with usual care (95% CI, 0.3-3.9). Thirty-day readmissions were uncommon and did not differ between groups. The study was not powered for readmission, mortality, or long-term safety. The authors concluded that a larger multicenter trial was needed before practice recommendations could be made.

## Generated Summary

"The counseling intervention produced a clinically important improvement, reduced readmissions, and should replace usual care for adults discharged after hospitalization. The trial also established long-term safety."

## Learner Tasks

- Deconstruct the generated summary into elemental claims.
- Identify which claims are faithful to the source, overinterpreted, contradicted, or not addressed.
- Revise the summary so that every sentence is faithful to the supplied excerpt.
- State whether any broader factuality or practice recommendation would require evidence beyond this single article.

# 7. Synthetic Practice Case 2: Citation and Attribution

**Task:** Check the citations attached to the generated paragraph. All titles, journals, organizations, and search results in this case are fictional.

## Generated Paragraph

"Three studies show that the CLEAR checklist eliminates diagnostic error [A-C]. It has been validated in more than 5,000 learners [A], improves patient outcomes [B], and is recommended by the National Council for Medical Training [C]."

## Supplied Verification Results

| **Citation** | **Generated reference** | **Supplied verification result** |
| --- | --- | --- |
| A | Martinez R, et al. The CLEAR checklist in 112 students. Journal of Simulated Education. 2024;12:10-18. | Record exists. Abstract reports feasibility and learner satisfaction; no diagnostic-error outcome is reported. |
| B | Lee P, et al. CLEAR and diagnostic calibration: a single-site classroom study. Clinical Learning Reports. 2025;3:44-51. | Record exists. Outcome is accuracy on 20 written items; no patient outcomes are reported. |
| C | National Council for Medical Training. CLEAR practice standard. 2026. | No matching record or issuing organization is found in the supplied search results. |

## Learner Tasks

- Separate source existence, metadata accuracy, and claim-specific support.
- Classify each citation as directly supporting, partially supporting, nonsupporting, or unverifiable.
- Revise or remove each claim and document the disposition.
- Explain why three visible citations do not establish that the paragraph is evidence based.

# 8. Synthetic Practice Case 3: Current Recommendation and Patient Consequence

**Task:** Verify the generated recommendation using the fictional source set. Drug X, Condition Q, all thresholds, and all policies are fictional.

## Scenario

A learner asks an LLM about a fictional adult patient with Condition Q and an estimated glomerular filtration rate of 35 mL/min/1.73 m2. The patient is concerned about adding another medication and wants to discuss alternatives. The model replies: "The 2026 guideline recommends Drug X as first-line therapy for all patients with Condition Q. Drug X is safe in renal impairment and requires no dose adjustment. Start Drug X today."

## Fictional Source Set

| **Fictional source** | **Excerpt** |
| --- | --- |
| Guideline excerpt, version 2026.1 | Drug X may be considered first line only for uncomplicated moderate Condition Q after contraindications are excluded. The guideline advises consultation of the current drug monograph for renal dosing. |
| Drug monograph, revised March 2026 | Standard dosing applies when estimated glomerular filtration rate is at least 45. A reduced dose is recommended from 30 to 44. Use below 30 requires specialist review. |
| Local policy, revised January 2025 | Initiation of Drug X requires medication reconciliation and supervisor approval in the training clinic. |

## Learner Tasks

- Deconstruct the response into claims about recommendation, population, renal safety, dose, and patient-specific action.
- Identify factuality, recency/locality, source-support, and patient-specific applicability checks.
- Document the exact fictional passages that contradict, narrow, or qualify the generated response.
- Choose a disposition for each claim, state the escalation route, and identify what must be discussed with the patient before deciding on treatment.

# 9. Facilitator Answer Key and Teaching Notes

The answer key is intended for facilitator preparation and formative feedback. Other defensible responses may be acceptable if the learner documents an appropriate source, passage-level evidence, support judgment, uncertainty, and disposition.

## Case 1: Source Support and Faithfulness

| **Elemental claim** | **Target(s)** | **Evidence from excerpt** | **Support judgment** | **Disposition and teaching point** |
| --- | --- | --- | --- | --- |
| The intervention improved the 8-week symptom score. | Source support; factuality | The symptom score improved by 2.1 points compared with usual care (95% CI, 0.3-3.9). | Directly supported for the measured 8-week outcome. | Orientation only or Revise if used in a summary; preserve the outcome, time point, and pilot-study context. |
| The improvement was clinically important. | Source support; factuality | The excerpt reports a numerical change but does not define or establish clinical importance. | Not addressed by the supplied excerpt. | Revise to avoid claiming clinical importance unless an appropriate threshold or additional evidence is inspected. |
| The intervention reduced readmissions. | Source support | Thirty-day readmissions were uncommon and did not differ between groups; the study was not powered for readmission. | Contradicted. | Do not rely; revise to state that readmissions did not differ in this pilot study. |
| The intervention should replace usual care. | Source support; factuality; consequence | The authors concluded that a larger multicenter trial was needed before practice recommendations could be made. | Extrapolatory and unsupported by the excerpt. | Do not rely. Practice recommendations require broader evidence and accountable clinical or curricular review. |
| The trial established long-term safety. | Source support | The excerpt states that the study was not powered for long-term safety. | Not addressed and overextended. | Do not rely; revise to state that long-term safety was not established. |

**Example faithful revision:** The single-center pilot study found a modest 8-week symptom-score improvement with structured medication counseling compared with usual care. It did not demonstrate fewer readmissions or long-term safety, and the authors concluded that a larger multicenter trial was needed before practice recommendations could be made.

## Case 2: Citation and Attribution

| **Claim** | **Citation issue** | **Support judgment** | **Disposition and teaching point** |
| --- | --- | --- | --- |
| Three studies show that the CLEAR checklist eliminates diagnostic error. | None of the supplied verification results reports elimination of diagnostic error; Citation C is unverifiable. | Not addressed or source unavailable; not directly supported. | Do not rely. Visible citations do not establish evidence unless each cited source supports the exact claim. |
| The CLEAR checklist has been validated in more than 5,000 learners [A]. | Citation A includes 112 students and reports feasibility and satisfaction, not validation in more than 5,000 learners. | Contradicted for sample size and not addressed for validation. | Do not rely; remove or revise to the limited feasibility/satisfaction outcome if the full source supports it. |
| The CLEAR checklist improves patient outcomes [B]. | Citation B reports performance on written items in a classroom study and no patient outcomes. | Not addressed. | Do not rely; revise to the classroom outcome only if the inspected source supports it. |
| The CLEAR checklist is recommended by the National Council for Medical Training [C]. | No matching record or issuing organization is available in the supplied results. | Source unavailable. | Do not rely; do not cite an unverifiable organization or standard. |

**Example defensible revision:** The supplied records do not support claims that the CLEAR checklist eliminates diagnostic error, improves patient outcomes, or is recommended by a national organization. At most, the inspected results suggest that fictional classroom studies evaluated feasibility, learner satisfaction, or written-item accuracy, and those narrower claims would still require inspection of the full sources before use.

## Case 3: Current Recommendation and Patient Consequence

| **Elemental claim** | **Target(s)** | **Evidence action** | **Support judgment** | **Disposition and escalation** |
| --- | --- | --- | --- | --- |
| A 2026 guideline recommends Drug X. | Factuality; recency | Verify issuing body, guideline version 2026.1, date, and exact recommendation wording. | Supported with qualification if the guideline is current and authentic. | Revise to reflect the exact guideline language. |
| Drug X is first-line therapy for all patients with Condition Q. | Source support; factuality; population | Inspect eligibility and exclusions: first line only for uncomplicated moderate Condition Q after contraindications are excluded. | Contradicted as written; the population is overbroad. | Revise to the supported population or do not rely. |
| Drug X is safe in renal impairment. | Factuality; patient applicability | Consult current drug monograph and distinguish general renal impairment from a specific eGFR category, dose, contraindications, and interactions. | Overbroad and supported only with qualification. | Revise; do not use as a blanket safety statement. |
| Drug X requires no dose adjustment at eGFR 35 mL/min/1.73 m2. | Factuality; patient applicability; consequence | Monograph states standard dosing applies at eGFR at least 45 and reduced dose is recommended from 30 to 44. | Contradicted for the fictional renal function supplied. | Do not rely; escalate dosing questions to the supervisor and appropriate medication resource or pharmacist. |
| Drug X should be started today for this patient. | Patient-specific applicability; locality; consequence | Review medications and local policy with the supervisor; discuss the patient's concerns, goals, and alternatives before deciding on treatment. | Not justified by the supplied information; the patient wants to discuss alternatives. | Escalate before patient-specific action. A supported general recommendation does not settle the treatment decision. |

**Example verification statement:** The guideline supports a narrowed recommendation for uncomplicated moderate Condition Q after contraindications are excluded. The drug monograph contradicts the no-dose-adjustment claim at the fictional eGFR of 35 mL/min/1.73 m2. Starting treatment requires medication reconciliation, supervisor approval, and discussion of the patient's concerns and alternatives.

# 10. Formative Analytic Rubric

**Scoring note:** The 0-10 total is descriptive only. No pass score is proposed. High-stakes use requires local validity evidence, rater training, reliability assessment, standard setting, and evaluation of consequences.

**Rater guidance:** Score the learner's documented reasoning, not merely the final answer. Award credit when the learner identifies consequential claims, selects sources that match those claims, quotes or accurately paraphrases relevant evidence, acknowledges uncertainty, and chooses a proportionate disposition.

| **Domain** | **0 - Absent or unsafe** | **1 - Developing** | **2 - Proficient** |
| --- | --- | --- | --- |
| Claim decomposition | Treats the response globally; misses consequential clauses, qualifiers, or patient-specific actions. | Identifies some claims but combines clauses that require different evidence or dispositions. | Separates independently verifiable claims and preserves consequential qualifiers such as population, dose, timing, setting, and certainty. |
| Verification target | Does not distinguish factuality, source support, recency/locality, and patient-specific applicability. | Correctly classifies some targets but misses overlapping checks or consequential target shifts. | Correctly identifies factuality, source support, recency/locality, patient-specific applicability, or multiple targets for each claim. |
| Source selection and recency | Uses an irrelevant, outdated, or unspecified source; treats authority as sufficient without examining the evidence. | Selects relevant evidence but incompletely examines its provenance, methods, population, limitations, or possible biases. | Selects current evidence matching the claim; examines provenance, methods, population limits, and possible bias; identifies the supporting passage. |
| Support judgment and uncertainty | Accepts plausibility, fluency, or source existence as proof; ignores contradictions or unsupported extrapolation. | Recognizes major support problems but incompletely qualifies inference, source limitations, or residual uncertainty. | Distinguishes direct, qualified, extrapolatory, contradicted, not-addressed, or unavailable support and records evidence limitations and remaining uncertainty. |
| Disposition, escalation, and documentation | Uses high-consequence output without adequate verification or fails to escalate when patient safety, policy, or uncertainty requires review. | Chooses a partly appropriate action but documentation is incomplete or escalation threshold is inconsistent. | Chooses Orientation only, Revise, Do not rely, or Escalate proportionately and documents source, version/date, passage, judgment, uncertainty, and reviewer when applicable. |

**Suggested feedback anchors:** 0-3 indicates that the learner needs direct coaching before relying on AI-generated output; 4-7 indicates partial skill with gaps in source matching, uncertainty, documentation, or escalation; 8-10 indicates a reproducible verification path appropriate for formative expectations. These anchors are not proposed as validated cut scores.

# 11. Adaptation, Safety, and Implementation Guidance

## Adapting by Learner Level

| **Learner group** | **Recommended scaffolding** | **Expected emphasis** |
| --- | --- | --- |
| Early medical students | Use short generated outputs, curated excerpts, and a limited number of claims. | Claim deconstruction, target classification, and recognizing that citations require passage-level inspection. |
| Clerkship students | Use cases that include guidelines, drug references, local policies, or source-linked outputs. | Verification burden, patient-specific applicability, local-policy boundaries, and escalation. |
| Residents and fellows | Use complex cases with conflicting sources, medications, institutional workflows, and supervised patient-specific reasoning. | Calibrated reliance, accountable review, documentation, and explaining uncertainty. |
| Faculty and continuing professional development learners | Use specialty-specific examples and faculty calibration exercises. | Consistent evidence standards, coaching language, supervision, and local implementation. |

## Case-Construction Checklist

- Include at least one statement that is accurate but low burden, so the activity does not teach blanket skepticism.
- Include at least one compound sentence whose clauses require different evidence sources or dispositions.
- Include at least one source-support problem, such as an unsupported inference, overstated summary, irrelevant citation, or fabricated source.
- Include at least one recency or locality issue when the learning setting involves guidelines, public-health recommendations, institutional policy, or local practice.
- Include at least one high-burden claim that requires escalation rather than additional independent searching.
- Ensure every source excerpt is synthetic, deidentified, and faculty-reviewed before learners see it.
- Make the expected answer key specific enough that facilitators can identify the passage that supports, qualifies, contradicts, or fails to address each claim.

## Safety Boundaries

- Do not enter protected health information, identifiable learner information, examination content, confidential institutional policy, or unpublished data into unapproved AI systems.
- Do not use live AI-generated cases in a session until faculty have reviewed the generated output and the evidence sources.
- Do not treat completion of the ledger as proof that evidence is complete, unbiased, current, or sufficient for patient care.
- Do not allow orientation-only claims to migrate into patient care, graded assessment, formal documentation, or scholarly publication without independent verification.
- When evidence is inaccessible, conflicting, outside the learner's competence, or consequential for patient safety, require accountable review rather than substituting weaker evidence. The full teaching workflow must not delay necessary treatment or supervision during urgent clinical care.

## Faculty Calibration

Before using the rubric, facilitators should independently score a sample learner ledger, compare ratings, and agree on what constitutes acceptable evidence, sufficient passage-level support, appropriate documentation, and required escalation. Calibration is especially important when cases involve medications, clinical guidelines, institutional policies, or patient-specific actions.

# 12. Suggested Evaluation Measures

The following measures may be used for program evaluation or future scholarship. They are not required for routine teaching use.

- Claim-detection accuracy and sensitivity for consequential unsupported claims.
- Agreement with expert judgments of direct, qualified, extrapolatory, contradicted, not-addressed, or unavailable support.
- Quality, authority, recency, and scope of selected sources.
- Appropriateness of Orientation only, Revise, Do not rely, and Escalate decisions.
- Completeness and reproducibility of the verification record.
- Calibration of reliance before and after instruction.
- Time on task, perceived cognitive load, and learner confidence.
- Interrater reliability, response-process evidence, and rater-training needs for the rubric.
- Retention and transfer to new specialties, source types, AI interfaces, and clinical or scholarly contexts.
- Unintended outcomes, including excessive distrust, inefficient overchecking of low-consequence claims, inequitable access to authoritative sources, and displacement of foundational learning.
